# Unearthing the global, regional, and national epidemiological landscapes of high BMI-driven ischemic heart disease burden in youth: Trends, determinants, and future trajectories

**DOI:** 10.1371/journal.pone.0344774

**Published:** 2026-03-19

**Authors:** Kejie He, Haitao Wang

**Affiliations:** 1 The Quzhou Affiliated Hospital of Wenzhou Medical University, Quzhou People’s Hospital, Quzhou, Zhejiang Province, China; 2 The school of Clinical Medical Sciences, Southwest Medical University, Luzhou, Sichuan, China; Dow University of Health Sciences, PAKISTAN

## Abstract

**Background:**

Cardiovascular diseases, including ischemic heart disease (IHD), are leading causes of mortality and morbidity worldwide. High body mass index (BMI) is a major contributing factor. This study aimed to comprehensively analyze the geographic distribution, socioeconomic trends, and age-period-cohort effects of the IHD burden attributable to high BMI to inform targeted interventions.

**Methods:**

Researchers utilized data from the Global Burden of Disease Study 2021 (GBD 2021) to examine the mortality, incidence, and disability-adjusted life years (DALYs) of IHD related to high BMI among 20–49 year-olds across 204 countries and territories. Joinpoint regression, age-period-cohort modeling, and decomposition analyses were employed to assess the heterogeneous epidemiological patterns across sociodemographic contexts.

**Results:**

The findings revealed stark geographical disparities, with Central America, the Caribbean, North Africa, and parts of the Middle East and Southeast Asia experiencing the most pronounced IHD burden. Across socioeconomic levels, low sociodemographic index (SDI) countries exhibited persistent increases, while high-middle and high SDI nations showed signs of stabilization or decline. The age-period-cohort analysis uncovered heterogeneous patterns, with the epidemiological transition progressing more rapidly in lower-SDI settings. Decomposition analysis indicated that epidemiological changes and population growth were the dominant drivers of the rising burden in less developed regions.

**Conclusions:**

This comprehensive analysis elucidates the geographic distribution and socioeconomic differences in the IHD burden attributable to high BMI, providing crucial evidence to guide the development of tailored public health interventions. Context-specific strategies are needed to address the persistent upward trends in resource-limited settings while consolidating the gains made in more developed countries to achieve equitable and sustainable cardiovascular health improvements.

## Introduction

Cardiovascular diseases, including ischemic heart disease (IHD), pose a significant global health challenge, responsible for a substantial burden of mortality and morbidity worldwide [1,2]. In recent decades, the prevalence of modifiable risk factors, such as high body mass index (BMI), has emerged as a major contributor to the growing burden of IHD [3,4], particularly among young adults and adolescents [5].

High BMI is a well-established risk factor for the development of cardiovascular diseases, including IHD [6,7]. Some settings have experienced a more pronounced increase in the IHD burden associated with high BMI, while others have shown more favorable epidemiological trends [5,8]. These diverging patterns underscore the complex interplay of social, economic, and environmental factors that shape the epidemiological transition in different contexts [9,10].

Understanding the geographical variations and sociodemographic trends in the IHD burden attributable to high BMI is crucial for informing targeted interventions and guiding policy decisions to address this pressing public health issue [11,12]. Comprehensive analyses that elucidate the complex, multifaceted nature of this challenge can provide valuable insights to enhance the development and implementation of evidence-based strategies to mitigate the growing burden [13,14].

This study aims to comprehensively analyze the global, regional, and local dynamics of the IHD burden attributable to high BMI among young adults and adolescents aged 20–49 years. Using advanced epidemiological modeling and decomposition techniques, the researchers seek to provide insights into the evolving patterns, identify the drivers of the observed trends, and inform the development of tailored, context-specific public health strategies to address this growing public health challenge.

## Methods

### Data retrieval

This retrospective study leveraged comprehensive data from the Global Burden of Disease Study 2021 (GBD 2021) to investigate the disease burden of IHD attributable to high BMI. The researchers obtained estimated deaths and disability-adjusted life years (DALYs) for IHD, across both sexes and 204 countries and territories [15,16].

Cardiovascular diseases, including IHD, were classified according to the International Classification of Diseases 10th edition (ICD-10) in the GBD 2021 database. This analysis specifically focused on the burden of IHD linked to high BMI among young adults and adolescents aged 20–49 years, spanning the period from 1990 to 2021.

To extract data from the GBD database, we used the publicly accessible Global Health Data Exchange (GHDx) query tool, which allows users to select disease categories, risk factors, age groups, geographical units, and temporal ranges. After specifying IHD as the outcome and high BMI as the risk factor, we downloaded age-specific and age-standardized mortality and DALY rates for all included countries. The extracted datasets were provided by GBD as modeled estimates based on standardized analytical pipelines incorporating multiple data sources and Bayesian statistical modeling.

To enable meaningful comparisons across diverse populations, the researchers utilized age standardization to adjust each country’s prevalence rate. Additionally, the SDI of the 204 countries was incorporated into the analysis, with countries classified into five distinct categories ranging from low to high SDI.

This comprehensive data source and analytical approach provided a robust foundation to examine the geographic distribution, socioeconomic trends, and epidemiological patterns of the IHD burden attributable to high BMI within the 20–49 age group. Age standardization was performed using the direct method. This method involves calculating age-specific rates for each country and then applying these rates to the standard population to compute standardized rates. The formula used is: Standardized Rate = Σ(Age-specific Rate_i × Standard Population Proportion_i).

In this study, age standardization was conducted using the GBD world standard population, which is a synthetic, stable demographic structure created to ensure comparability across regions and time. This standard population is characterized by a balanced distribution across age groups and is widely used in GBD studies to eliminate confounding due to differences in population age structures.

### Statistical analysis

We employed rigorous statistical methodologies to comprehensively analyze epidemiological trends in IHD burden associated with elevated BMI. Joinpoint regression analysis was utilized to assess the trends in mortality, incidence, and prevalence across the study countries.

To ensure the validity of the trend analysis, the researchers excluded countries with missing or zero values in any year. The standard error (SE) was calculated using a standardized formula that incorporated the upper and lower bounds of the 95% confidence intervals obtained from the Global Burden of Disease (GBD) data.

The study further quantified the average annual percentage change (AAPC) and its corresponding 95% confidence intervals, employing geometric weighting to demonstrate the trends across various SDI regions. The researchers compared the magnitude of AAPC to zero, considering a non-significant difference as indicative of a stable trend.

The selection of statistical methods was guided by sample size considerations rather than the segmentation of AAPC. We employed a statistical distribution aligned with the sample size and data characteristics: the t-distribution was used when the sample size was relatively small, while the normal (z) distribution was applied for larger sample sizes, ensuring the most appropriate parametric approach for confidence interval estimation.

Moreover, we provided a more explicit rationale for selecting these methods. Joinpoint regression was used because it enables the detection of changes in temporal trends without imposing a priori assumptions about inflection points, making it particularly suitable for long-term epidemiological series such as the GBD dataset. The APC and AAPC indices quantify both local and overall temporal trends, which aligns with our objective of assessing disease burden dynamics across SDI regions. The use of parametric confidence intervals for APC/AAPC further ensured statistical stability given the modeled nature of GBD estimates.

All statistical analyses were performed using Joinpoint and R software version 4.2.0 for Windows, with the significance level set at p < 0.05. This rigorous analytical framework provided a comprehensive assessment of the epidemiological trends in IHD burden attributable to high BMI across the study population.

## Results

### Geographical variations in the IHD burden attributable to high BMI among young adults and adolescents worldwide

**Fig 1:**
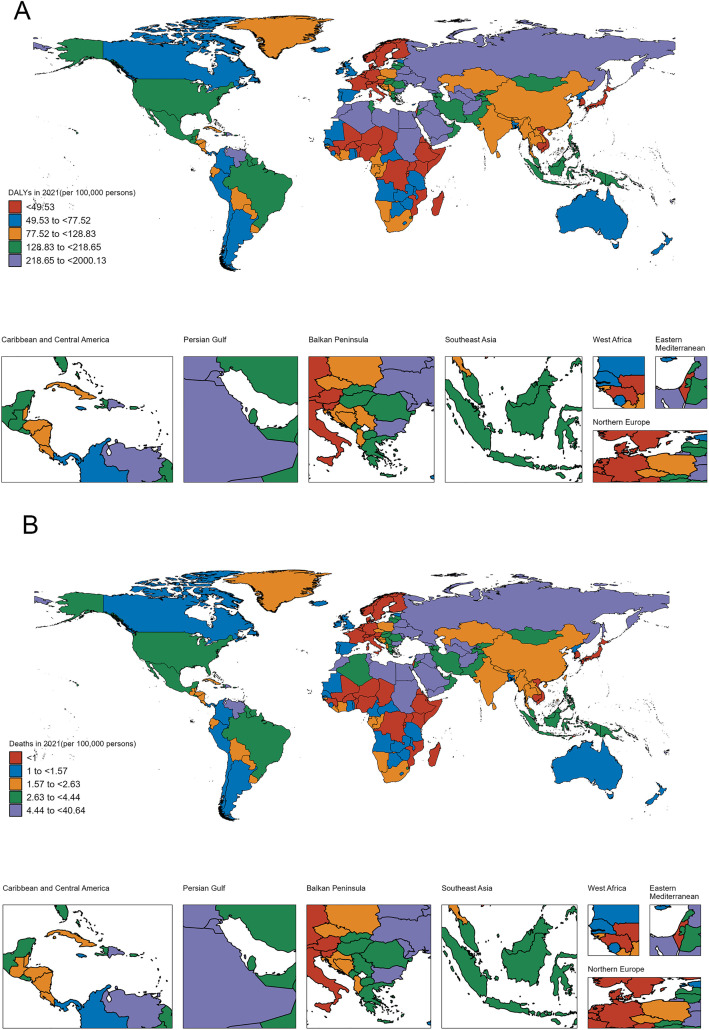
Geographical variations in the IHD burden attributable to high BMI among young adults and adolescents worldwide. Republished from the Resource and Environment Science and Data Center (https://www.resdc.cn/) under a CC BY license, with permission from the Institute of Geographic Sciences and Natural Resources Research, Chinese Academy of Sciences, original copyright [2025] **(A)** Global distribution of DALYs due to IHD per 100,000 population among individuals aged 20-49 years. **(B)** Global distribution of mortality rates due to IHD per 100,000 population among individuals aged 20-49 years.

The two world maps in Fig 1 depict the global burden of IHD attributable to high BMI among individuals aged 20–49 years old.

Map A illustrates the geographical distribution of DALYs due to IHD per 100,000 population. The countries are shaded according to five discrete color-coded categories, ranging from the lowest DALY rate (<49.53) to the highest (>218.65). This spatial visualization highlights the substantial regional disparities in the health impact of high BMI on young adults and adolescents.

The regions with the most pronounced IHD burden, represented by the darkest shades of purple, include Central America, the Caribbean, North Africa, and parts of the Middle East and Southeast Asia. Conversely, countries in Western Europe, North America, and Australasia exhibit relatively lower DALY rates for this age group (Fig 1A).

Map B presents a similar geographic breakdown, but focusing on the mortality rates due to IHD per 100,000 population. The color scale again denotes five distinct categories, this time ranging from the lowest death rate (<1.0) to the highest (>4.44).

The spatial patterns observed in the mortality map largely mirror those seen in the DALY map, with the highest death rates concentrated in Central America, the Caribbean, North Africa, and parts of the Middle East and Southeast Asia. Regions like Western Europe, North America, and Australasia continue to exhibit relatively lower IHD mortality among young adults and adolescents (Fig 1B).

These contrasting maps provide a comprehensive overview of the global epidemiological trends and geographical disparities in the burden of IHD associated with high BMI in the 20–49 age group. The findings underscore the urgent need for targeted public health interventions and policy actions to address this growing threat to cardiovascular health, particularly in the most affected regions.

### Diverging epidemiological trends in high BMI-Attributable IHD burden across sociodemographic contexts

This Fig presents a comprehensive set of time-series analyses examining the trends in DALYs attributable to high BMI from 1990 to 2020, both globally and across different SDI levels.

**Fig 2:**
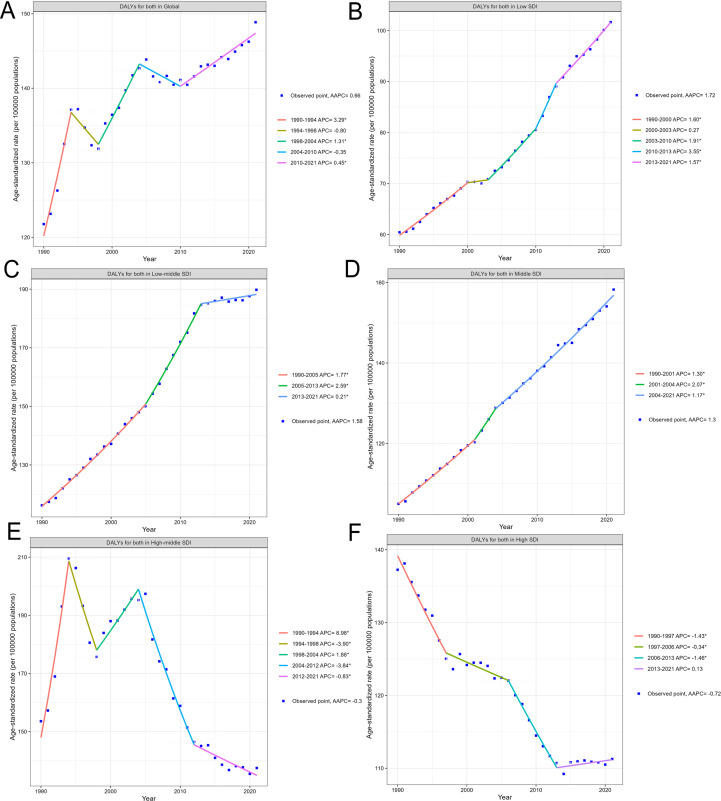
Diverging epidemiological trends in high BMI-Attributable IHD burden across sociodemographic contexts. **(A)** Global trends in DALYs attributable to high BMI from 1990 to 2021. **(B-F)** DALY trends across different SDI levels from 1990 to 2021, including low SDI, low-middle SDI, middle SDI, high-middle SDI, and high SDI countries.

The overall global DALY trend for both sexes is depicted. The data points represent the observed values, while the line illustrates the forecasted trajectory. The DALY rate has steadily increased from approximately 120 per 100,000 population in 1990 to around 147 per 100,000 in 2021, indicating a substantial rise in the global burden of high BMI-associated ischemic heart disease (Fig 2A)

The DALY trends in low SDI and low-middle SDI countries show a relatively slower but consistent increase, rising from around 60–100 per 100,000 and 115–190 per 100,000, respectively, over the study period. This suggests a gradual epidemiological transition in these resource-limited settings (Figs 2B and 2C)

For middle SDI countries, the DALY rate has more rapidly increased from roughly 105 to nearly 160 per 100,000, indicating a faster epidemiological transition in these middle-income settings (Fig 2D)

The DALY trends for high-middle SDI countries exhibit a complex pattern. These countries show an initial increase in DALY rates from the 1990 to the 1994, followed by a subsequent decline in the later years. The observed data points and the model-based projection capture this non-linear trend, with the AAPC indicating a decrease over the full study period (Fig 2E).

Similarly, the high SDI countries demonstrate a continued decrease in DALY rates, but at a faster pace compared to other nations. Importantly, the AAPC for high SDI countries also shows a decreasing trend, suggesting that the overall long-term burden may be stabilizing or even declining in the most socioeconomically developed settings. (Fig 2F).

Collectively, the insights gained reveal a nuanced picture, with lower-SDI countries still experiencing increasing DALY rates, while higher-SDI nations show signs of stabilization or even decline in the long-term AAPC. This diverging pattern across the socioeconomic spectrum underscores the complex nature of the epidemiological transition and highlights the need for targeted, context-specific public health interventions and policy decisions. The decreasing AAPC trends in high-middle and high SDI countries suggest that effective prevention and management strategies may be taking hold in these more developed settings, providing valuable lessons that can be adapted and scaled to address the persistent burden in lower-resourced regions.

### Heterogeneous ge-Period-Cohort trends in high BMI-Attributable IHD burden across sociodemographic contexts

This Fig presents the results of an Age-Period-Cohort analysis examining the annual percent change in ischemic heart disease burden attributable to high body mass index, stratified by global and different SDI levels.

**Fig 3:**
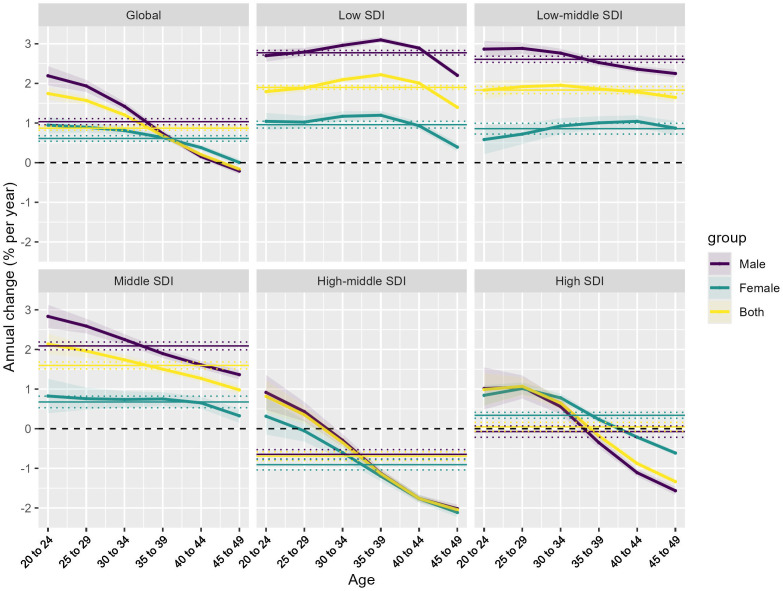
Age-period-cohort trends. Heterogeneous age-period-cohort trends in high BMI-Attributable IHD burden across sociodemographic contexts.

The top-left panel shows the overall global trends. The data exhibits an increasing annual percent change in the young people, peaking around the 20–24 and 25–29 year age ranges, followed by a decline. Examining the subsequent panels for the different SDI levels, distinct epidemiological patterns emerge: In the low SDI and low-middle SDI settings, the age-specific patterns show a relatively higher but consistent increase in the annual percent change for both males and females, with the male line consistently higher than the female line across the age range (Fig 3).

However, in the middle SDI countries, the age-specific annual percent change trends demonstrate a clear decrease, particularly in the older 40–44 and 45–49 age groups. This indicates a more favorable epidemiological transition in these middle-income nations. The high-middle SDI and high SDI countries also exhibit a declining trend in the annual percent change in the older age groups, suggesting a stabilization or even reversal of the high BMI-attributable ischemic heart disease burden in these more developed settings.

The horizontal solid lines in each panel denote the net drift, reflecting the overall temporal trend in disease burden as expressed by the AAPC. The estimated net drift values, together with their corresponding confidence intervals, illustrate both global and SDI-specific patterns, thereby enhancing the interpretation of epidemiological shifts across different socioeconomic contexts. These findings underscore the heterogeneous nature of the high BMI-attributable ischemic heart disease burden, with distinct age-specific and gender-specific patterns observed across the SDI levels. The diverging trends, particularly the favorable declines seen in the middle, high-middle, and high SDI countries, suggest that effective prevention and management strategies may be taking hold in these more developed settings, providing valuable lessons that can be adapted to address the persistent burden in lower-resourced regions.

### Age effects on the epidemiological transition of high BMI-Attributable IHD burden across sociodemographic contexts

The age-specific rate (per 100,000 population) exhibits an increasing trend across all SDI levels, with some variations in the magnitude of the increase.

**Fig 4:**
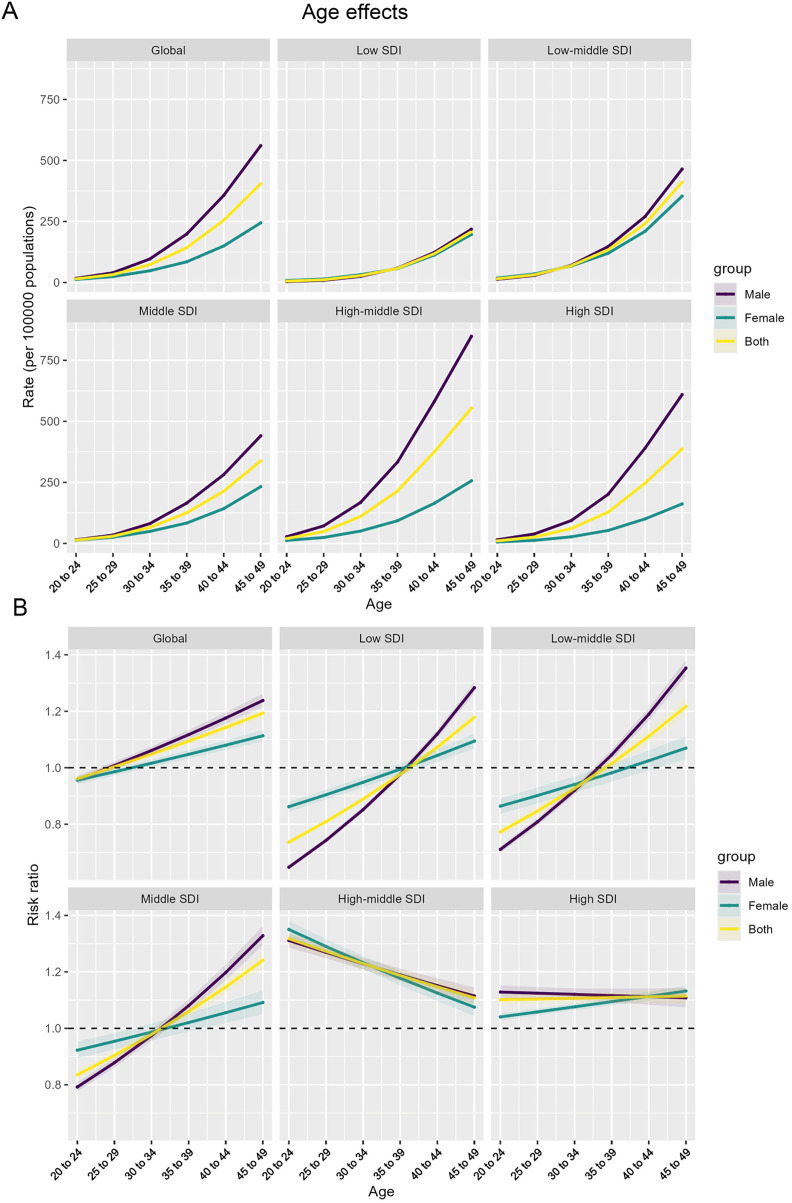
Age effects on the epidemiological transition of high BMI-Attributable IHD burden across sociodemographic contexts. **(A)** Age-specific rates (per 100,000 population) of ischemic heart disease attributable to high body mass index. **(B)** Relative risk ratios across age groups, providing insights into age, period, and cohort effects.

In the global, low SDI, and low-middle SDI settings, the rate shows a relatively slower but consistent increase for both males and females, with the male line consistently higher than the female line across the age range. However, the high-middle SDI and high SDI countries display a more pronounced increase in the age-specific rate, compared to the other SDI levels. This indicates a steeper epidemiological transition in these more developed settings (Fig 4A).

The relative risk (RR) ratios, which provide insights into the age, period, and cohort effects, show a diverging pattern across the SDI levels. For the global, low SDI, and low-middle SDI countries, the RR ratios consistently increase across the age groups, with males exhibiting higher values compared to females. In contrast, the high-middle SDI and high SDI countries demonstrate a decreasing trend in the RR ratios. This suggests a more favorable epidemiological transition in these more developed settings, potentially driven by effective prevention and management strategies (Fig 4B).

The Age-Period-Cohort model reveals a complex epidemiological landscape of body mass index-attributable ischemic heart disease, with age effects demonstrating distinctive patterns across SDI levels. By decomposing age-specific rates and relative risk ratios, our analysis uncovers a nuanced gradient: low and low-middle SDI regions exhibit consistent risk increments with male rates predominantly higher, while high-middle and high SDI countries display a more sophisticated epidemiological transition characterized by steeper initial increases followed by potential stabilization or risk reversal. These age-dependent variations, analyzed through the APC model’s multidimensional lens, highlight how cardiovascular disease burden is dynamically modulated by age, reflecting intricate interactions between demographic structures, healthcare interventions, and generational health transformations across different socioeconomic contexts.

### Divergent period and cohort effects on the epidemiological transition of high BMI-Attributable IHD burden across sociodemographic contexts

The period effects represent the influence of time-varying factors, such as changes in disease diagnosis, screening, and treatment, on the ischemic heart disease burden attributable to high body mass index across different SDI levels.

**Fig 5:**
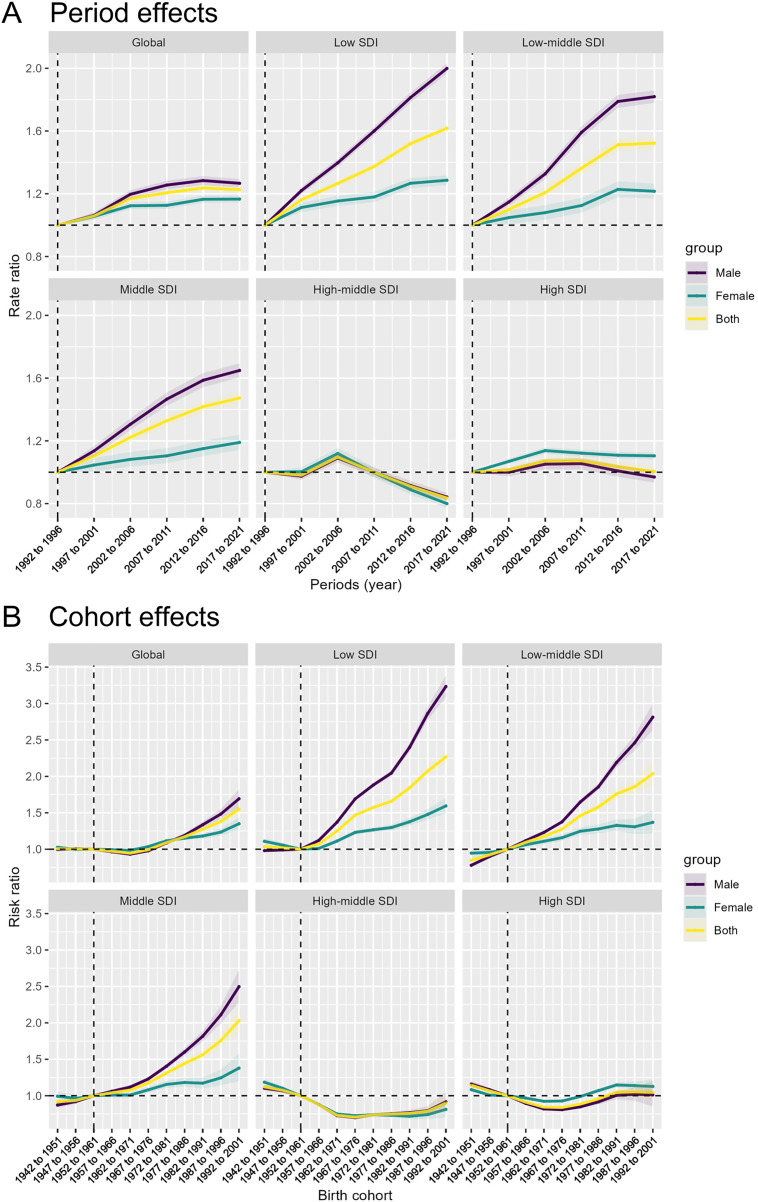
Divergent period and cohort effects on the epidemiological transition of high BMI-Attributable IHD burden across sociodemographic contexts. **(A)** Period effects, representing the influence of time-varying factors on the ischemic heart disease burden. **(B)** Cohort effects, capturing the differences in disease burden across birth cohorts.

The global trend shows a gradual increase in the period effects over time. This pattern is also observed in the low SDI, low-middle SDI, and middle SDI countries, indicating a worsening burden due to the influence of period-specific factors in these settings. However, the high-middle SDI and high SDI countries exhibit a more complex trend, with the period effects initially increasing but then declining in the later time periods. This suggests that the more developed settings may have implemented effective interventions or experienced improvements in disease management strategies that have mitigated the burden over time (Fig 5A).

The cohort effects capture the differences in disease burden across birth cohorts, reflecting the varying degrees of exposure to risk factors among different generations. Across all SDI levels, the cohort effects show an increasing trend, with males generally exhibiting higher values compared to females. This suggests that the newer birth cohorts are experiencing a greater burden of ischemic heart disease attributable to high body mass index. The patterns are more pronounced in the low SDI, low-middle SDI, and middle SDI countries, indicating a widening generational gap in the disease burden within these resource-limited and middle-income settings.

In contrast, the high-middle SDI and high SDI countries demonstrate a less steep increase in the cohort effects, suggesting a more favorable trajectory in the more developed settings, potentially due to targeted interventions and improved health care access (Fig 5B).

These findings on the period and cohort effects provide valuable insights into the heterogeneous epidemiological transition of high body mass index-attributable ischemic heart disease burden across diverse sociodemographic contexts. The insights can inform tailored public health strategies to address this growing global challenge.

### Heterogeneous drivers of health burden transitions across sociodemographic contexts: a decomposition analysis

At the global level, epidemiological changes appear to have the largest positive contribution to the increase in health burden, followed by population growth, while population aging plays a relatively smaller role.

However, when analyzing the results across different SDI levels, a more heterogeneous pattern emerges:

In the high SDI and high-middle SDI regions, the three factors – aging, population, and epidemiological change – exhibit a more balanced contribution to the changes in health burden, indicating a complex epidemiological transition process in these more developed settings.

**Fig 6:**
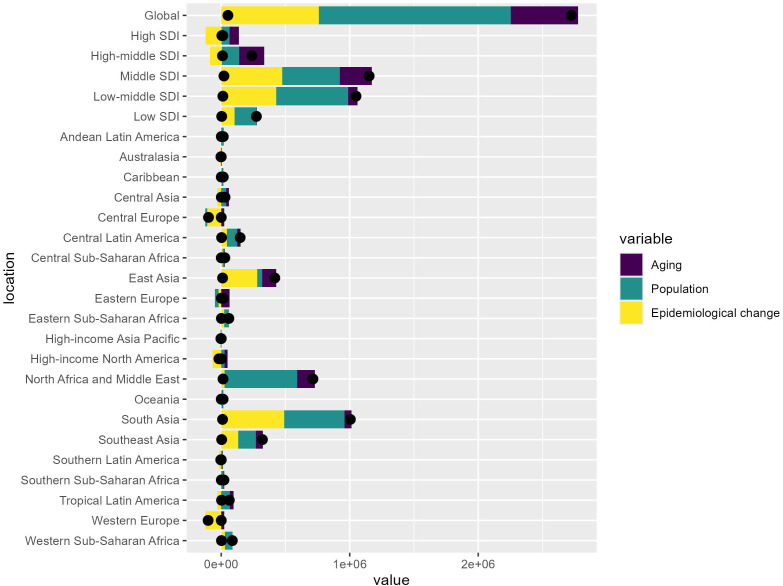
Decomposition analysis. Heterogeneous drivers of health burden transitions across sociodemographic contexts.

In contrast, the low SDI, low-middle SDI, and middle SDI regions show a greater dominance of epidemiological changes and population growth in driving the increase in health burden. This suggests a more rapid and uneven epidemiological transition occurring in these less developed areas (Fig 6).

Overall, this Decomposition Analysis provides valuable insights into the varying drivers of health burden changes across different socioeconomic contexts. These insights can inform the development of targeted public health interventions and policies to more effectively address the complex and diverse health challenges experienced globally.

By quantifying the relative contributions of aging, population dynamics, and epidemiological shifts, this analysis can guide priority-setting and resource allocation decisions to tackle the evolving health burden in a contextually appropriate manner.

### Projected global disease burden to 2044: widening gender disparities in total cases and age-standardized rates

**Fig 7:**
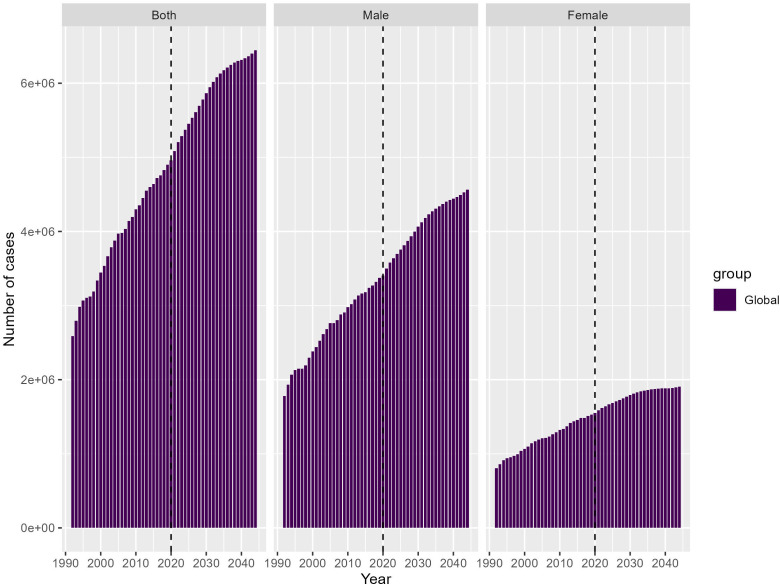
Projected GBD to 2044. Widening gender disparities in total cases and age-standardized rates.

Both sexes: The total number of cases for both sexes is projected to increase substantially from around 2.5 million in 1990 to over 6 million by 2044. Males: The total number of cases among males shows a steeper increase compared to females, rising from around 2 million in 1990 to nearly 4 million by 2044. Females: The total number of cases among females increases at a slower pace, from around 1.5 million in 1990–2 million in 2044 (Fig 7).

These projections provide a sobering outlook on the future trajectory of the global disease burden, highlighting the importance of implementing comprehensive, evidence-based strategies to address the rising trends, particularly among the male population. Tailored approaches that consider the distinct epidemiological patterns between genders will be crucial in mitigating the anticipated disease burden and promoting more equitable health outcomes worldwide.

## Discussion

The stark geographical disparities in the IHD burden attributable to high BMI among young adults and adolescents underscore the need for targeted, context-specific interventions [17,18]. Regions with the most pronounced burden, like Central America and North Africa, face multifaceted challenges that exacerbate the impact of high BMI. For instance, countries in these regions often struggle with food insecurity, limited access to healthcare, and a lack of public health infrastructure, which can contribute to the high prevalence of high BMI and associated cardiovascular diseases [19,20]. Moreover, historical dietary transitions, rapid urbanization, and the increasing penetration of ultra-processed foods in many low- and middle-income regions have further accelerated the rise in obesity-related cardiovascular risks [21,22]. Climate factors, patterns of physical labor, and environmental stressors also shape the geographic heterogeneity observed in our analysis, illustrating that biological, behavioral, and structural determinants jointly influence the distribution of IHD burden across different parts of the world [23].

In contrast, the lower disease burden observed in Western Europe and North America suggests these developed settings have implemented successful strategies to mitigate the adverse effects of high BMI. Countries like the United Kingdom, Germany, and Canada have implemented comprehensive national policies to promote healthy eating, increase physical activity, and improve access to weight management services within the primary care system [24,25]. These targeted efforts, combined with healthcare infrastructure improvements and public awareness campaigns, likely contributed to the more favorable epidemiological trends in the high-middle and high SDI settings [26,27]. Higher SDI regions also benefit from more stable food environments, higher health literacy, earlier detection through routine screenings, and stronger systems for chronic disease management, all of which help reduce the progression of high BMI into clinically significant cardiovascular disease [28].

The diverging epidemiological patterns across sociodemographic settings highlight the heterogeneous nature of this challenge. The persistent increases in DALY rates among low and low-middle SDI countries reflect entrenched barriers, including limited healthcare access, ongoing nutritional transitions, and sociocultural factors that shape dietary and physical activity behaviors [29,30]. Conversely, the more favorable trajectories in high-middle and high SDI countries indicate that comprehensive, systems-level approaches to prevention and management may be effective in more developed settings [31,32]. These contrasting trends call for tailored, equity-focused strategies addressing the unique drivers within each context. In lower SDI regions, broader structural determinants—such as economic instability, limited transportation infrastructure, gender inequality, and restricted access to cardiovascular medications—further amplify the observed discrepancies [33]. In contrast, higher SDI settings show reduced burden not only because of stronger healthcare systems but also because of preventive policies embedded in schools, workplaces, and community environments, enabling a sustained reduction in modifiable risk factors [34].

The nuanced age-period-cohort analysis further illuminates the heterogeneous epidemiological transition, with the persistent increases in younger age groups and widening generational gaps in less developed settings underscoring the urgent need to prioritize primordial and primary prevention targeting the youth [35,36]. In contrast, the more favorable trends in higher SDI countries suggest successful interventions for older age groups, providing valuable lessons for adaptation, such as the implementation of workplace wellness programs and community-based lifestyle interventions [37,38]. The observed socioeconomic gradient in ischemic heart disease burden reveals profound systemic inequities beyond epidemiological trends. Low SDI countries experience persistent increases due to structural challenges including limited healthcare infrastructure, inadequate preventive interventions, and restricted access to nutritional education. While high SDI nations have developed sophisticated prevention strategies, low SDI regions struggle with fundamental health system constraints [39]. These disparities create a cyclical economic burden, where limited resources impede cardiovascular prevention, subsequently increasing healthcare costs and reducing workforce productivity. Addressing these challenges requires comprehensive, context-specific strategies that integrate economic development, health system strengthening, and targeted social policies, moving beyond traditional medical interventions to tackle the root causes of cardiovascular health inequities.

The decomposition analysis highlights the dominant role of epidemiological changes and population growth in driving the rising IHD burden in lower SDI regions, suggesting the need for comprehensive approaches addressing social, behavioral, and environmental determinants. Exploring successful policies and innovations in higher SDI settings, such as the implementation of urban planning initiatives to promote active transportation and the development of multisectoral collaborations to address the social determinants of health, can inform the adaptation of effective strategies globally [40,41]. Furthermore, population aging proceeds differently across SDI strata, with some low-SDI regions experiencing rapid demographic transitions without concurrent health system development, exacerbating the cardiovascular consequences of high BMI [42]. This demographic mismatch magnifies the contribution of population growth to overall disease burden in resource-limited settings.

Lastly, the sobering gender disparities in projected IHD burden demand targeted, gender-responsive interventions to ensure equitable access to preventive services and address sociocultural barriers faced by young men. Successful examples include the integration of gender-specific approaches within primary care settings, the development of community-based programs that engage men in health promotion, and the implementation of workplace policies that support work-life balance and promote physical activity [43,44]. Biological differences—such as variations in fat distribution, hormonal regulation, and inflammatory response—contribute to sex-specific susceptibility to high BMI-related cardiovascular damage [45]. In addition, behavioral and sociocultural factors, including lower healthcare-seeking behavior among men, higher exposure to occupational hazards, and differing norms surrounding physical activity and diet, also play a significant role in shaping the gender gap in IHD burden. These intertwined biological and social determinants highlight the need for gender-tailored preventive frameworks.

While recognizing the diverse socioeconomic landscapes across different regions, we propose a stratified approach to addressing high BMI-related IHD burden. For low SDI countries, resource-efficient interventions include community-based education programs on nutrition and physical activity, leveraging mobile health technologies for low-cost health messaging, training community health workers in basic cardiovascular risk assessment, implementing cost-effective screening programs using simple anthropometric measurements, and developing school-based nutrition and physical activity curricula. These strategies emphasize adaptive implementation through collaboration with local community leaders, utilizing peer-education models, developing culturally sensitive health communication, and creating scalable, low-cost intervention models. Middle and high SDI countries can focus on more comprehensive approaches, such as integrating cardiovascular risk screening into primary healthcare systems, developing national prevention programs, implementing workplace wellness initiatives, and supporting policy interventions targeting food environments. Cross-cutting recommendations include developing flexible intervention models adaptable to local contexts, prioritizing cost-effectiveness and sustainable implementation, emphasizing prevention over treatment, fostering interdisciplinary collaboration, and continuously evaluating and adapting intervention strategies.

Recognizing the complexity of Western trends in cardiovascular health, our study acknowledges the need for a more comprehensive examination of contributing factors beyond targeted interventions for healthy eating and exercise. While our research provides valuable insights into IHD among young adults, we recognize the importance of a broader contextual analysis. Specific IHD intervention strategies for young adults should include comprehensive screening protocols, such as early lipid profile assessments, genetic risk factor evaluations, and non-invasive cardiovascular imaging techniques like coronary calcium scoring. Moreover, targeted interventions could encompass lifestyle modification programs, stress management techniques, and personalized cardiovascular risk counseling. The socioeconomic landscape, including urban-rural disparities, educational attainment, and systemic health inequalities, plays a crucial role in shaping individual and population-level cardiovascular health behaviors. Although we have developed robust BMI-related recommendations, the discussion would benefit from a more in-depth examination of age-specific IHD prevention approaches, integrating psychological, behavioral, and precision medicine perspectives. Future research should therefore adopt a more holistic approach, combining clinical interventions with comprehensive environmental, social, and individual-level factors to develop more targeted and effective cardiovascular health strategies for young adults.

Our study’s primary focus on BMI-attributable IHD burden necessarily simplifies a complex physiological relationship. Physical inactivity and smoking represent critical elements that may modify the BMI-IHD relationship. These factors can independently contribute to cardiovascular risk, interact synergistically with high BMI, and amplify the underlying physiological mechanisms of cardiovascular disease. In lower SDI countries, the combination of high BMI, limited physical activity, and potentially higher smoking prevalence may create a more pronounced cardiovascular risk profile. While providing valuable population-level insights, our analysis cannot fully capture individual-level risk variations, intricate interactions between multiple risk factors, or comprehensive individual cardiovascular risk profiles. These limitations underscore the importance of comprehensive approaches to cardiovascular health that extend beyond BMI measurement alone. Future research should develop more integrated risk assessment models, explore the interactive effects of multiple cardiovascular risk factors, and utilize advanced analytical techniques to unpack complex risk pathways.

In conclusion, this analysis offers insights into the global, regional, and sociodemographic dynamics underlying the IHD burden attributable to high BMI. The findings call for context-specific, evidence-based strategies that address the complex, intertwined drivers of this growing public health crisis. Collaborative efforts to strengthen health systems and promote equity can reduce unacceptable disparities in cardiovascular health [46,47]. By leveraging successful interventions from high-performing settings and adapting them to local contexts, policymakers and public health practitioners can work towards a more equitable and sustainable future for cardiovascular health worldwide. While our study provides valuable insights into the global burden of ischemic heart disease associated with body mass index, several methodological limitations must be acknowledged. The cross-sectional nature of the GBD 2021 dataset prevents establishing causal relationships and may mask individual-level variations in cardiovascular risk. Standardized BMI categories fail to capture nuanced physiological differences across diverse populations, particularly regarding body fat distribution and metabolic characteristics. Data quality and reporting inconsistencies across different countries potentially introduce systematic biases, with low-to-middle-income regions potentially having less robust health surveillance mechanisms. Furthermore, our analysis could not comprehensively integrate critical confounding factors such as socioeconomic status, dietary patterns, and genetic predispositions. These limitations underscore the complexity of global cardiovascular health research and highlight the need for more sophisticated, population-specific approaches to understanding the relationship between body composition and ischemic heart disease. Our study recognizes that the exclusive use of ICD-10 codes may introduce potential data continuity challenges. The transition between classification systems can create subtle but significant variations in diagnostic categorization, potentially impacting the comprehensive representation of historical medical data. These variations might arise from differences in disease definitions, coding specificity, and diagnostic criteria between ICD-9 and ICD-10 systems.

## Conclusions

This comprehensive study analyzed the global, regional, and local dynamics of the ischemic heart disease burden attributable to high body mass index. The findings revealed stark geographical disparities, with Central America, the Caribbean, North Africa, and parts of the Middle East and Southeast Asia experiencing the most pronounced burden among young adults and adolescents. Across socioeconomic levels, low SDI countries exhibited persistent increases, while high-middle and high SDI nations showed signs of stabilization or decline. The age-period-cohort analysis uncovered heterogeneous patterns, with the epidemiological transition progressing more rapidly in lower-SDI settings. Decomposition analysis indicated that epidemiological changes and population growth were the dominant drivers of the rising burden in less developed regions. These insights provide crucial evidence to guide the development of tailored public health interventions to address this growing cardiovascular health challenge. Context-specific strategies are needed to tackle the persistent upward trends in resource-limited settings while consolidating the gains made in more developed countries to achieve equitable and sustainable improvements in cardiovascular health worldwide.
